# pH-Responsive Metal–Organic Framework for Targeted Delivery of Fungicide, Release Behavior, and Sustainable Plant Protection

**DOI:** 10.3390/molecules29225330

**Published:** 2024-11-12

**Authors:** Shuzhen Yang, Fulin Lü, Li Wang, Sinan Liu, Zhisai Wu, Yanqin Cheng, Fan Liu

**Affiliations:** College of Plant Protection, Shanxi Agricultural University, Jinzhong 030801, China

**Keywords:** zeolitic imidazolate framework-8, thiabendazole, pH-responsive, sustainable plant protection, leaching loss

## Abstract

A smart and environmentally friendly pesticide system was developed that could respond to environmental stimuli while mitigating environmental risks. In this study, thiabendazole (Thi), an effective fungicide, was loaded onto zeolitic imidazolate framework-8 (ZIF-8) using the impregnation method to fabricate a pH-responsive nano hybrid delivery system (Thi@ZIF-8). The results demonstrated that Thi@ZIF-8 had a rhombic dodecahedral morphology and a loading capacity of approximately 25%. Notably, the amount of Thi released from Thi@ZIF-8 at a pH of 5.0 reached 79.54%, which was higher than that at pH 7.0 and 9.0, for 251 h. Such pH-responsive release characteristics of Thi@ZIF-8 were probably related to the pH-dependent structure stability of ZIF-8. The release mechanism of Thi@ZIF-8 conformed to non-Fickian diffusion. Additionally, Thi@ZIF-8 showed a higher control efficacy against *B. cinerea* compared with Thi alone. Importantly, the ZIF-8 carrier could effectively reduce the leaching loss of Thi in soil and showed no negative effects on the three varieties of tomato seedlings, implying good biocompatibility. This work provides a novel and eco-friendly approach to control B. cinerea effectively that has great potential in modern sustainable agriculture.

## 1. Introduction

Pesticides play an integral role in safeguarding crop growth, warding off pests and diseases, and ensuring the yield of agricultural products [1,2,3,4,5]. However, 70% of pesticides are lost and degraded after spraying due to factors such as wind-driven dispersal, rainwater erosion, volatilization, and soil leaching [6]. Therefore, the effective utilization rate is less than 1%. The majority of compounds entering the environment further influence the nutrient cycle and food chain [7,8,9,10,11].

Therefore, modern pesticides are becoming more “green”, with an increasing focus on high efficiency, low toxicity, low dosage, and low residue. Reducing pesticide usage from the source is highly important to offset environmental chemical pollution. Stimulation-responsive controlled-release systems based on environmental stimuli, such as light [12,13,14,15,16], heat [17,18,19,20], pH [21,22,23,24], and enzymes [25,26,27,28], can achieve the on-demand release of chemical compounds across both time and space dimensions. Such systems can significantly reduce pesticide usage, improve utilization efficiency, and control pesticide losses [29].

Metal–organic frameworks (MOFs) are a kind of porous material with various types of organic linker ligands via coordination interactions [30,31]. The zeolite imidazole ester framework material ZIF-8 is one of the most common and widely used MOF materials and is obtained by coordinating metal Zn^2+^ ions with dimethylimidazole [32,33]. In recent years, the ZIF-8 material has been widely studied in the fields of biomedicine and nanomedicine and has been considered a good drug carrier with many advantages, such as simple synthesis, high porosity, high drug loading rate, and pH-responsive degradation [34,35]. Under low pH conditions, the coordination between the zinc ions and the imidazole rings is disrupted [36], leading to the gradual degradation of the ZIF-8 structure and the release of the drugs. ZIF-8 can also be used to construct pH-responsive drug carriers [37] and apply these ZIF-8-regulated pesticides on fungus-infected crops. The acidic substances secreted by the fungal pathogen result in an acidic microenvironment at the infection site [38,39] and effective pathogen control, thereby achieving the goal of targeting the infection area by specific degrading at the optimal site and the release of the pesticide.

This study used the pH-sensitive porous material ZIF-8 as a carrier of thiabendazole (Thi), employed a simple impregnation method to prepare Thi@ZIF-8, and determined the optimal preparation conditions for obtaining a high-drug-loading controlled-release agent. The antibacterial effects of Thi@ZIF-8 on tomato grey mold were then investigated in vitro and in vivo. Thi@ZIF-8 was successfully optimized to reduce costs while maintaining a high level of efficiency and adhering to environmental protection standards, promoting the sustainable development of modern agriculture.

## 2. Results and Discussion

### 2.1. Morphological Observation

**Figure 1:**
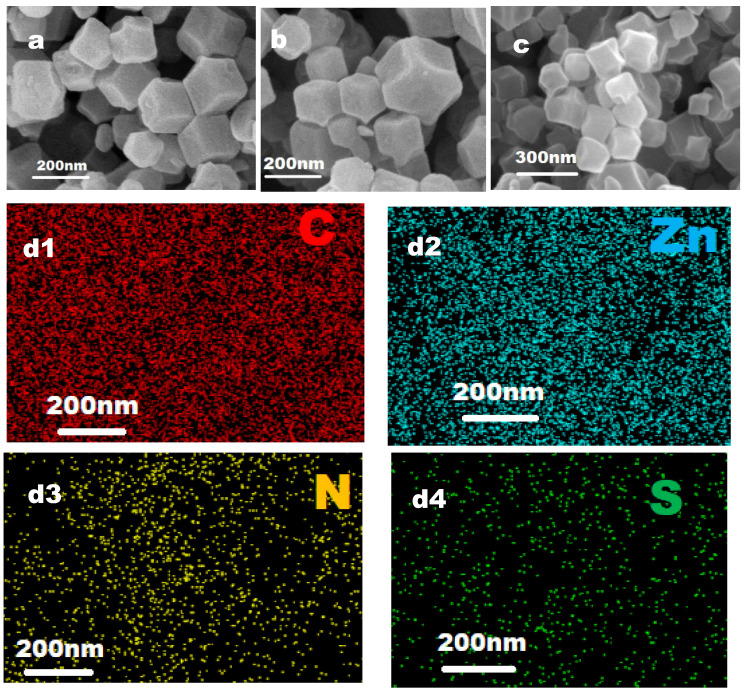
SEM images of (**a**) ZIF-8 and (**b**) Thi@ZIF-8. (**c**–**d4**) Elemental mapping images corresponding to (**d1**) C, (**d2**) Zn, (**d3**) N, and (**d4**) S elements of Thi@ZIF-8.

ZIF-8 nanoparticles were prepared via self-assembly between Zn ions and 2-methylimidazole. A SEM image (Figure 1a) shows the dodecahedral morphology [40] of ZIF-8, and the particle size was around 200 nm. From the scanning electron microscope image of Thi@ZIF-8 particles shown in Figure 1b, it can be seen that the crystal shape of Thi@ZIF-8 was regular, the grain size distribution was relatively uniform, the surface was smooth, and there was a slight agglomeration phenomenon between particles. To investigate the elemental distribution of Thi@ZIF-8, elemental maps were obtained, as shown in Figure 1(c,d1–d4). The results illustrated that these elements of Zn, C, N, and S existed and were distributed in the shell of Thi@ZIF-8. The uniform distribution of Zn elements indicated the successful synthesis of ZIF-8 [14]. The S element belonging to Thi was uniformly distributed in ZIF-8, indicating the presence of Thi in Thi@ZIF-8 particles. However, the intensity of the S element was relatively weak, and this was the result of a low content of S in Thi@ZIF-8.

### 2.2. X-Ray Powder and FT-IR Spectra Analysis

**Figure 2:**
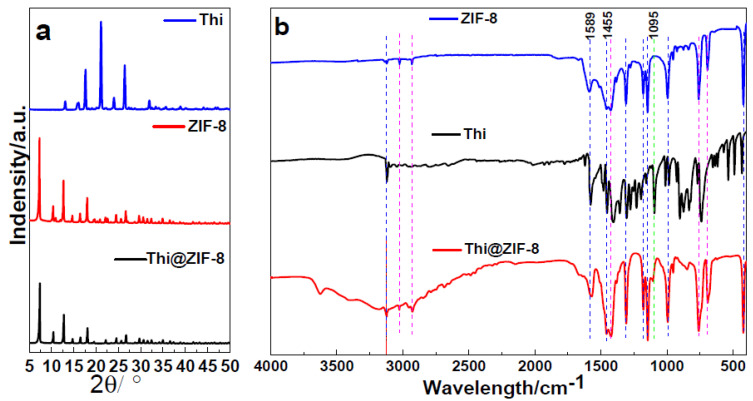
(**a**) XRD spectrum of ZIF-8, Thi, and Thi@ZIF-8. (**b**) Infrared spectra of ZIF-8, Thi, and Thi@ZIF-8. The blue line is a peak shown the same peaks in ZIF-8, Thi and Thi@ZIF-8, the purple line shown the same peaks in ZIF-8 and Thi@ZIF-8, the green line shown the unique peak in Thi and Thi@ZIF-8.

The crystallinity of the synthesized sample was evaluated using X-ray diffraction (XRD) analysis. The results indicated that ZIF-8, Thi, and Thi@ZIF-8 were highly crystalline with sharp diffraction peaks (Figure 2a). Distinctive characteristic peaks for ZIF-8 were evident at 2θ values of 7.38°, 10.42°, 12.76°, 14.74°, 16.48°, 18.07°, 22.17°, 24.55°, 26.72°, and 29.71°, which corresponded to the (011), (002), (112), (022), (013), (222), (114), (233), (134), and (044) crystal planes of pure ZIF-8, respectively. This pattern aligns closely with previously reported data [41]. Thi@ZIF-8 showed characteristic peaks consistent with those of ZIF-8, indicating that the loading process had no effect on the crystalline structure of ZIF-8 and that its crystal structure remained intact. Meanwhile, no diffraction peaks were observed from Thi, and the decrease in intensity values for Thi@ZIF-8 indicated that the Thi molecules were indeed encapsulated inside the ZIF-8 pores.

The FT-IR spectra of Thi, ZIF-8, and Thi@ZIF-8 are shown in Figure 2b. The obtained FT-IR spectra of ZIF-8 closely aligned with previously reported literature [42]. The peaks situated at 3120 cm^−1^, 3026 cm^−1^, and 2928 cm^−1^ were ascribed to the N-H, =C-H, and C-H asymmetric stretching vibrations, respectively. The peaks situated at 1589 cm^−1^ and 1445 cm^−1^ were attributed to the C=N stretching vibrations. The absorption peak was observed at 1178 cm^−1^, corresponding to the C–N asymmetric stretch, and 995 cm^−1^, attributable to the N-H bending vibrations. Furthermore, the absorption peak at 422 cm^−1^ was attributed to the stretching of Zn-N [43,44], indicating the successful synthesis of the ZIF-8. Many similar or identical peaks appeared in Thi and ZIF-8 (blue color) because Thi and ZIF-8 contain imidazole rings. After Thi loaded, intensity changes were observed in certain peaks of Thi@ZIF-8, and no new characteristic peaks appeared compared with before ZIF-8 loading. However, the peak at 1095 cm^−1^ was ascribed to the S-N stretching vibrations, as with the characteristic peak in Thi. Nevertheless, the stretching peaks of C=N of ZIF-8 and S-N of Thi were shifted from 1589 cm^−1^ and 1095 cm^−1^ to 1571 cm^−1^ and 1091 cm^−1^ of Thi@-8, respectively, which indicated that there may have been an interaction between Thi and ZIF-8. Therefore, Thi was successfully loaded by the ZIF-8.

### 2.3. XPS Analysis

**Figure 3:**
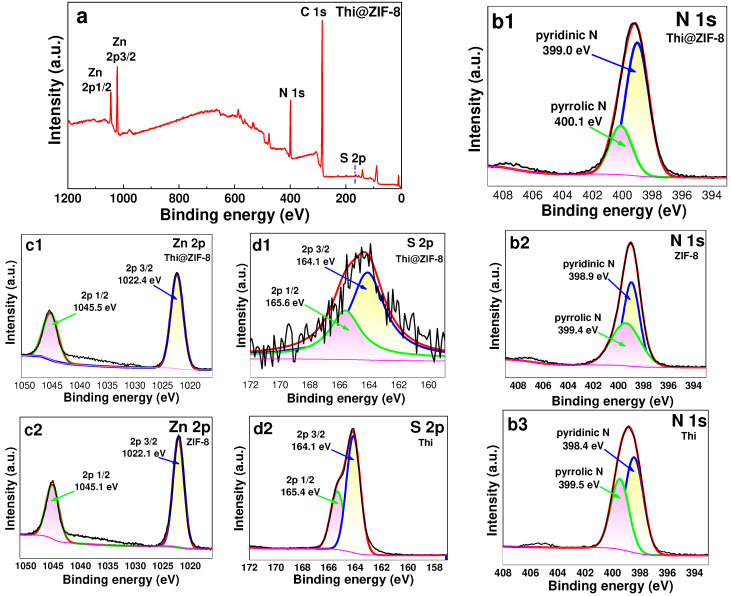
(**a**) Full XPS spectra of Thi@ZIF-8 and high-resolution XPS of Thi@ZIF-8. (**b1**) N 1s spectra in Thi@ZIF-8. (**b2**) N 1s spectra in ZIF-8. (**b3**) N 1s spectra in Thi. (**c1**) Zn 2p spectra in Thi@ZIF-8. (**c2**) Zn 2p spectra in ZIF-8. (**d1**) S 2p spectra in Thi@ZIF-8. (**d2**) S 2p spectra in Thi.

The elemental chemical composition and bonding types of the prepared Thi@ZIF were characterized by X-ray photoelectron spectroscopy (XPS), and the results are shown in Figure 3a. The survey spectra of Thi@ZIF-8 exhibited the characteristic elemental peak of ZIF-8, which was consistent with the reported literature [42]. In addition, there was an S 2p peak, indicating that Thi was successfully loaded onto ZIF-8. To study the interaction mechanism between Thi and ZIF-8, the samples were characterized via Zn 2p, S 2p, and N 1s analysis at high resolution. Figure 3(b1–b3) displays the N 1s XPS spectra, which yielded peaks at 398.9 and 399.4 eV, which were identified as the binding of pyridinic N and pyrrolic N in ZIF-8 [45] (Figure 3(b2)), respectively, and the corresponding peaks at 398.4 and 399.5 eV in Thi (Figure 3(b3)), respectively. Two peaks shifted to 399.0 and 400.1 eV in Thi@ZIF-8 (Figure 3(b1)), also indicating that N may play a role in the process of Thi loading [46]. As shown in Figure 3(c1,c2), the peaks at 1022.1 eV and 1045.1 eV were assigned to Zn 2p 3/2 and Zn 2p 1/2 in ZIF-8 (Figure 3(c2)), respectively. After Thi loading, both peaks increased and shifted to 1022.4 eV and 1045.5 eV (Figure 3(c1)), respectively, which indicated that there may have been interaction forces between Thi and Zn. As shown in Figure 3(d1,d2), the S 2p1/2 peak was shifted from 165.4 eV of Thi to 165.6 eV of Thi@-8, which indicated that S may play a role in the process of Thi loading.

### 2.4. Zeta Potentials and TGA Analysis

**Figure 4:**
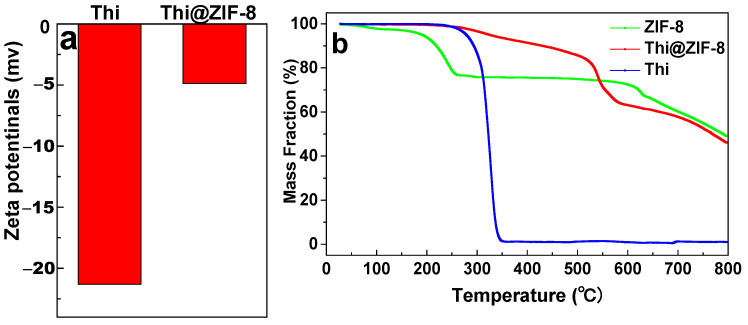
(**a**) The zeta potentials of Thi and Thi@ZIF-8. (**b**) Thermogravimetric analysis (TGA) curves of ZIF-8, Thi, and Thi@ZIF-8.

The zeta potentials of Thi and Thi@ZIF-8 are shown in Figure 4a. The zeta potential of Thi was −17.3 mV, whereas it became −4.87 mV after loading ZIF-8, which was attributed to the charge of ZIF-8 [47]. The change of surface charge indicated that Thi was loaded successfully, and one of the drivers was electrostatic adsorption. The results from SEM mapping, XRD, FTIR, and XPS also indicated that Thi loaded onto ZIF-8.

TGA is mainly used to analyze the thermal stability and decomposition behavior of materials. Figure 4b shows the Thi, ZIF-8, and Thir@ZIF-8 weight loss curves when heated from room temperature to 800 °C at a rate of 15 °C/min. The results revealed that the low weight loss rate of ZIF-8 at approximately 2.5% before 100 °C was possibly due to the evaporation of surface solvent molecules. The quality of ZIF-8 decreased by 20% between 100 °C and 260 °C, and this decrease may have been due to the carbonization of organic ligands on the surface of the carrier [48]. As the temperature continued to rise beyond 600 °C, the weight loss may have been caused by the collapse of the framework of ZIF-8. The weight loss rate of Thi was close to 100% from 250 °C to 350 °C, but Thi@ZIF-8 displayed an additional weight loss of approximately 5.7% within the same temperature range. The improved thermal stability of Thi below 600 °C was most likely due to its successful loading onto ZIF-8. The results showed that Thi-loading systems prepared using a carrier could effectively improve the thermal stability of Thi.

### 2.5. The Loading Efficiency of Thi onto ZIF-8

**Figure 5:**
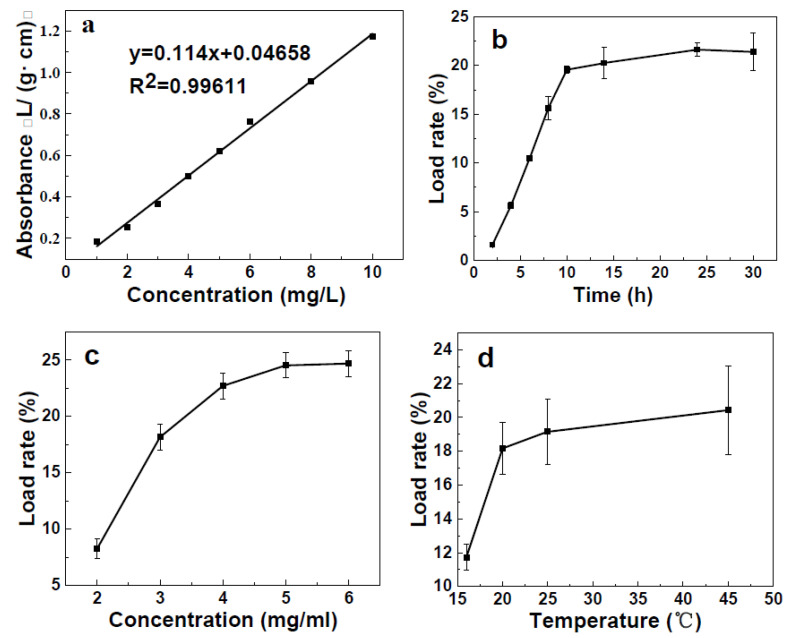
Single-factor experiment. (**a**) Standard curves of Thi. (**b**) Loading rates of ZIF-8 at different times. (**c**) Loading rates of ZIF-8 at different concentrations. (**d**) Loading rates of ZIF-8 at different temperatures.

To investigate the loading capacity of ZIF-8 at different conditions, a standard curve of Thi was established, as shown in Figure 5a. The curve indicated that the concentrations of Thi had a good linear relationship with the loading rate between 1 and 10 mg/L (R^2^ = 0.99611).

Figure 5b shows the effect of time on the loading of Thi onto ZIF-8. For these cases, the initial Thi concentration was 3 mg/mL, and a temperature of 20 °C was used. Additionally, the ZIF-8 dose was 0.0400 g in 20 mL of anhydrous ethanol. The loading rate of Thi reached up to 20.25% when the loading time was 14 h, after which, the loading rate remained relatively constant. This observation revealed that the adsorption of Thi was slow and that an equilibrium state was attained after 14 h of loading time. Considering the economics, a loading time of 14 h was chosen for the subsequent experiments.

Concentration was an important controlling parameter in all the loading processes. The other conditions were fixed at 10 h of loading time and a 20 °C loading temperature. The results showed significant changes in the loading capacities for the Thi concentration range from 2 to 6 mg/mL (Figure 5c). The highest loading capacity was about 25% at 5 mg/L, and a decrease in loading capacity was observed with decreasing concentration. When the concentration of Thi was greater than 5 mg/L, the loading rate of ZIF-8 showed almost no growth. Thus, 5 mg/L was considered the optimal concentration and was used in the subsequent steps of this study.

The loading rates of Thi onto ZIF -8 at different temperatures are shown in Figure 5d. The loading rate of Thi increased significantly below 25 °C and slowly at temperatures above 25 °C. For economic reasons, a temperature of 25 °C was chosen for the loading of Thi onto ZIF-8.

The optimal preparation conditions for Thi@ZIF-8 were 25 °C, 15 h, and 5 mg/mL, with a loading capacity of approximately 25%, which was higher compared with previous studies on pyraclostrobi@ZIF-8 (loading efficiency 10.8%) [48] and dinotefuran@ZIF-8 (loading efficiency 12.32%) [49]. A possible reason for this was that the Thi molecules were relatively small compared to other drugs and easily entered the pores of ZIF-8. The high loading capacity allowed for a significant reduction in the amount of ZIF-8 carrier required for each dose of pesticide delivery to just 50%. A high antibacterial effect was maintained while reducing the amount of ZIF-8 entering the environment.

### 2.6. Thi@ZIF-8 Release Analysis

**Figure 6:**
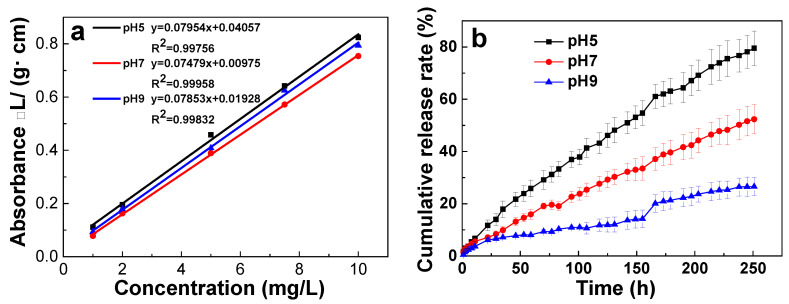
(**a**) Standard curves of Thi in PBS with different pH values. (**b**) Release rate of Thi@ZIF-8 at different pH values.

To examine the pH-driven response of Thi@ZIF-8 release behavior, standard curves of Thi were established in PBS adjusted to different pH levels (Figure 6a). The results indicated that the concentration of Thi had a good linear relationship with absorbance between 1 and 10 mg/L, with the regression R^2^ fit ranging from 0.99756 to 0.99958.

In vitro release experiments of Thi were performed under various pH conditions (5.0, 7.0, 9.0). The pH-responsive release behavior of Thi@ZIF-8 is illustrated in Figure 6b. Thi from Thi@ZIF-8 reached 79.54% at pH 5.0, while only 52.34% and 26.55% were released in neutral (pH 7.0) and alkaline (pH 9.0) conditions within 251 h. These results demonstrated an excellent acid-responsive controlled-release performance of Thi@ZIF-8. This pH-responsive behavior could be attributed to the pH-dependent structural stability of the ZIF-8 carrier. Figure 6b also reveals that the release cycle of Thi from Thi@ZIF-8 lasted up to 251 h, which was longer compared with pyraclostrobi@ZIF-8 (88.1% release quantity in 24 h) [48] and t β-CYP/ZIF-8 (98.2% release quantity in 120 h) [36]. The result indicated that Thi@ZIF-8 could keep the high antibacterial effect for a long time, and the frequency of pesticide application was reduced.

**Figure 7:**
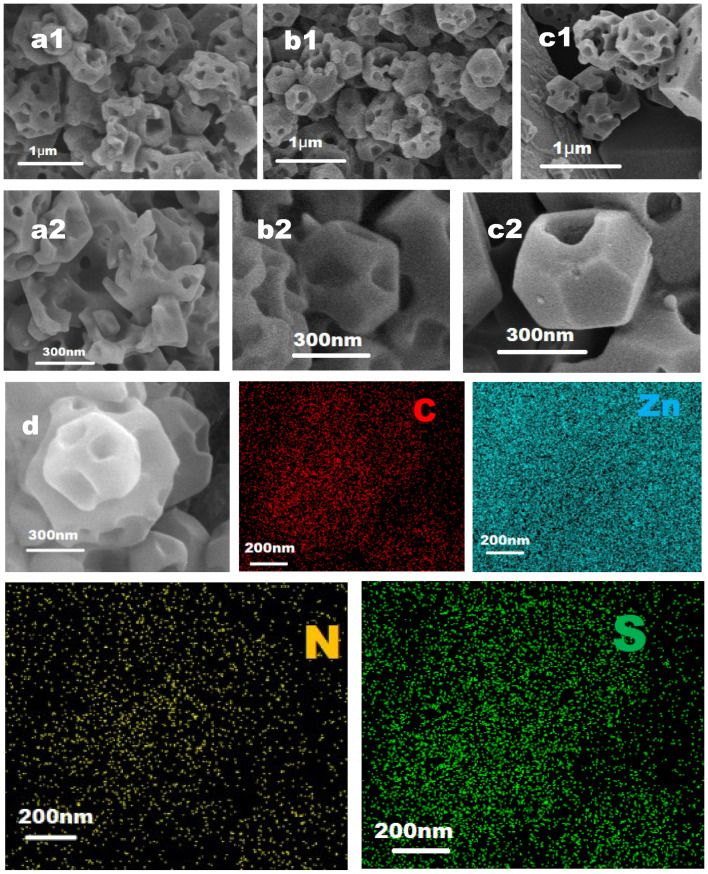
SEM images of Thi@ZIF-8 after release at different pH values ((**a1**,**a2**) pH 5.0; pH (**b1**,**b2**) 7.0; (**c1**,**c2**) pH 9.0). The SEM images (**d**) after release at a pH of 5.0 and elemental mapping images corresponding to C, Zn, N, and S elements of Thi@ZIF-8.

To better investigate the release process of Thi from ZIF-8, a SEM image was taken after release of 120 h, shown in Figure 7. As shown in Figure 1b, the surface of the Thi@ZIF-8 particles was smooth and without any holes. But, Figure 7(a1–c2) shows that the surface of ZIF-8 appeared with different depths and numbers of holes under different acid-base conditions. The particles of ZIF-8 had the most and deepest holes—and even began to disintegrate into fragments—under acid conditions (pH 5.0), followed by neutral (pH 7.0) and alkaline (pH 9.0) conditions. Therefore, it could be speculated that the release of Thi largely relied on the disintegration of ZIF-8. Under acidic conditions, the protonation disrupted the coordination between Zn^2+^ and the imidazole ring, which is consistent with previous studies [50,51,52]. Meanwhile, the (011) crystal face of ZIF-8 was more likely to be damaged, leading to the decomposition of the crystal structure [53,54]. With the dissociation of the crystal framework, Thi in ZIF-8 was gradually released. The stability of ZIF-8 under neutral conditions depended on the ratio of ZIF-8 to water and the contact time [55]. However, the structure of ZIF-8 after 120 h of release at a pH of 9.0 was different from that observed in previous studies [56]. It was possible that different preparation conditions affected the stability of ZIF-8. Thereby, the decomposition degree of ZIF-8 at pH 5.0, 7.0, and 9.0 dominated the release of Thi, which was consistent with the release behavior of Thi observed in other research. This showed that ZIF-8, as a carrier, achieved active controlled release of Thi in acidic environments. Given the unique acidic microenvironment generated by *B. cinerea*, Thi@ZIF-8 has great potential for targeted and sustained applications against *B. cinerea*.

The elemental distribution of Thi@ZIF-8 after release (pH 5.0) of 120 h was analyzed by mapping (Figure 7d). The results showed that the elements C, Zn, N, and S were also simultaneously detected in Thi@ZIF-8 after release. However, the intensity of the S element in Thi@ZIF-8 increased compared with before release (Figure 1(d4)). The probable reason was that more Thi was exposed and detected with the skeleton collapses that formed a hollow cavity. Additionally, it could be speculated that Thi entered the interior of the ZIF-8 pore channel, which improved the stability of Thi.

**Figure 8:**
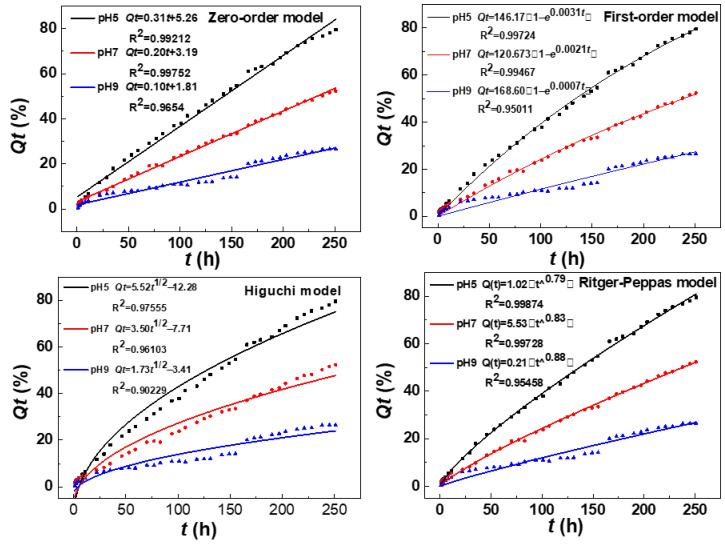
Kinetic model fitting curves for release profiles at different pH values.

To better understand the release behavior of Thi from Thi@ZIF-8, the release dynamic models were studied at different pH values (pH = 5.0, 7.0, and 9.0). The release curves were fitted using the zero-order, first-order, and Higuchi models, with the fitting results detailed in Table 1 and Figure 8. The release data of Thi from Thi@ZIF-8 at pH = 5.0, 7.0, and 9.0 were all best fit for the zero-order model, with a higher R^2^ (0.9654) than other models. This indicated that the release rate of Thi from Thi@ZIF-8 was constant or near-constant during the release cycle. In addition, the hydrophobicity of ZIF-8 prevented the premature or rapid release of Thi [57]. The zero-order model is an ideal model for the release of controlled-release formulations. In this study, the model implied that ZIF-8 is an excellent carrier for Thi. The Thi molecules in the Thi@ZIF-8 framework could be released as a controlled performance and continued to play a role in stably controlling *B. cinerea*. However, many release results were better fitted with the first-order model in previous studies of controlled-release formulations [23,36]. The first-order model fitted the release of the sustained-release platform. It indicated that the release rate was not constant and easily to experience “peak valley” fluctuations of pesticide concentration.

The controlled-release mechanism of Thi@ZIF-8 under different pH conditions was predicted by the value of the diffusion index (*n*) using the Ritger–Peppas model. The n value also influences desorption behavior (0 < *n* < 0.45 Fickian diffusion; 0.45 < *n* < 0.89 non-Fickian diffusion; *n* ˃ 0.89 skeleton dissolution mechanism) [58]. The n value of the Ritger–Peppas model at pH 5.0, 7.0, and 9.0 was 0.79, 0.83, and 0.88, respectively. All the Ritger–Peppas model n values were 0.45 < *n* < 0.89, which showed that Thi@ZIF-8 followed non-Fickian diffusion under different pH conditions. Similar results were found in previous research on release mechanisms [49,59]. Thus, the release process of Thi was controlled by the combined effect of the Fickian diffusion of Thi and the skeleton dissolution of ZIF-8, which was consistent with the results from SEM after release.

### 2.7. Leaching Transport of Thi@ZIF-8 in Soil

**Figure 9:**
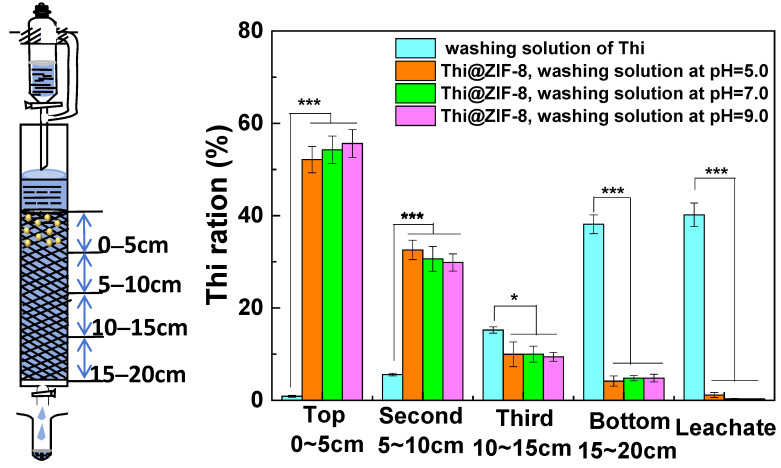
Distribution of Thi and Thi@ZIF-8 in the different sections of the soil column and the leachate. The data in the figure are the average of 3 repetitions. Statistically significant differences are indicated by * *p* < 0.05, and *** *p* < 0.001.

Generally, pesticides sprayed in the field are deposited on the target surface. However, they also infiltrate the soil and contaminate groundwater, which may cause a series of environmental risks. The distribution of Thi and Thi@ZIF-8 in soil was investigated using a soil column leaching method under laboratory conditions (Figure 9). The Thi molecules easily migrated from the top layer to the bottom of the soil column because these molecules can freely flow with water. Thi molecules were mainly detected from 10 to 20 cm in the soil column, and most molecules migrated into the 15–20 cm section (38.14%) or the leachate (40.17%). For Thi@ZIF-8, the Thi content in the leachate was significantly reduced. Thi molecules from Thi@ZIF-8 were still locked in the Thi@ZIF-8 framework. The Thi migration pattern was different between Thi and Thi@ZIF-8. As a result, the Thi from Thi@ZIF-8 was mainly distributed between 0 and 10 cm in the soil column, and most Thi was retained in the 0–5 cm section. A similar result was found in previous research [60]. In this case, because of the controlled-release method, the Thi@ZIF-8 had an advantage in protecting the tomatoes for a longer period and reducing the leaching loss and environmental pollution.

### 2.8. Plant Safety Evaluation

**Figure 10:**
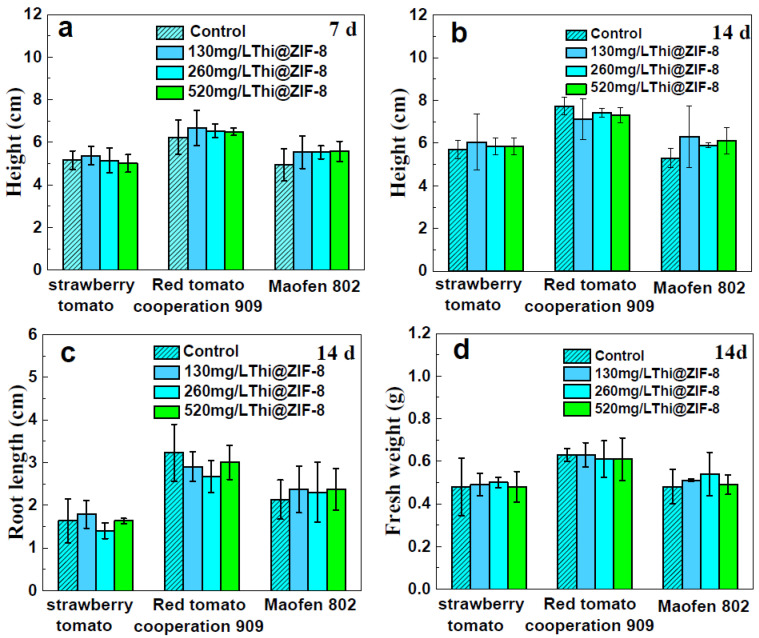
(**a**) Height of tomato seedlings treated with Thi@ZIF-8 after 7. (**b**) Heigh, (**c**) root length, and (**d**) fresh weight of tomato seedlings treated with Thi@ZIF-8 after 14 d.

Compared to traditional pesticide formulations, the carrier of ZIF-8 was also introduced into the environment together with the Thi, so its environmental safety needed to be taken into consideration. Previous study results illustrated that ZIF-8 exhibited benign biocompatibility in vitro and in vivo [49,61]. The safety of Thi@ZIF-8 nano particles in three different varieties of tomato seedlings was assessed by measuring fresh weight, plant height, and root length on the 7th and 14th days after spraying with different concentrations of Thi@ZIF-8 (130, 260, and 520 mg/L), as shown in Figure 10. The key growth indicators of tomato seedlings showed that plant height (Figure 10a,b), fresh weight (Figure 10c), and root length (Figure 10d) had no significant differences between the treatment and control groups. The above results implied that the ZIF-8 carriers had good biocompatibility and biosafety for tomato seedlings. This finding was in agreement with previous studies [62,63].

### 2.9. Fungicidal Activity

**Figure 11:**
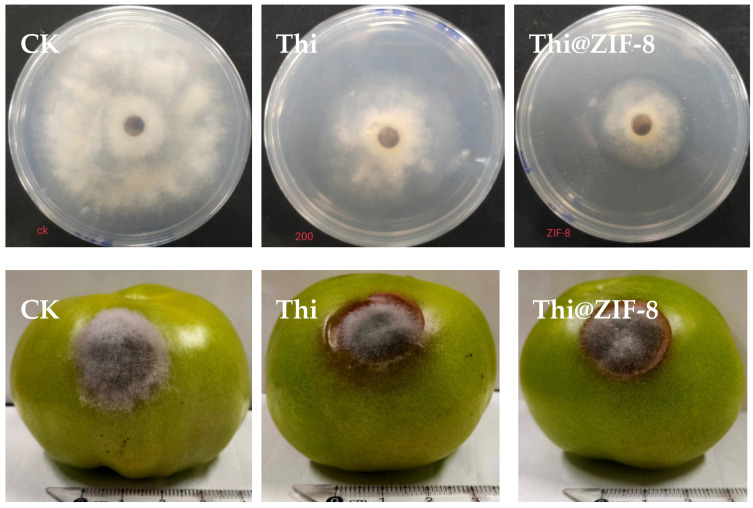
Fungicidal activity of Thi and Thi@ZIF-8 against *B. cinerea*.

*B. cinerea* is one of the most destructive diseases of tomato. Fungi such as *B. cinerea* secrete acidic substances, including oxalic acid, to accelerate the infection process in plants [64]. The resulting acidic microenvironment in our study promoted the dissolution of Thi@ZIF-8, triggering the release of Thi into the fungus-infected tomato. The fungicidal activity of Thi and Thi@ZIF-8 against *B. cinerea* was determined using the growth rate method, as shown in Figure 11 and Table 2. Under the concentration of 200 mg/L of the active ingredient Thi, Thi@ZIF-8 generated an inhibitory rate of *B. cinerea* of 59.07% ± 0.55% in the PDA, which was 30.17% higher than free Thi. Thi@ZIF-8 was sprayed over a tomato wound of imbibing *B. cinerea*; after seven days, the effectiveness of Thi@ZIF-8 on the fungus was 32.58% ± 5.8% (Figure 11 and Table 2), which was 9.82% higher than the treatment with Thi. These results indicated that Thi@ZIF-8 was more effective than Thi against *B. cinerea*. The ZIF-8 carrier also exhibited fungicidal activity against *B. cinerea*, likely owing to the fungicidal activity of Zn^2+^ ions contributing to the enhanced bioactivity of Thi@ZIF-8, which was in agreement with the results of previous research [61]. Additionally, Thi@ZIF-8 likely reduced the degradation of the active ingredient, thereby prolonging the effectiveness of Thi in Thi@ZIF-8, and exhibited superior fungicidal activity. By combining pH-responsive ability and security, Thi@ZIF-8 has great potential in sustainable agriculture to improve the utilization of Thi and reduce environmental pollution. This work contributes to the development of precise and safe pesticide delivery systems.

## 3. Materials and Methods

### 3.1. Materials and Reagents

Zinc acetate (Zn(CH_3_COO)_2_·2H_2_O, ≥99%) and absolute ethanol (C_2_H_5_OH) were obtained from Tianjin Tianli Chemical Reagent Company Limited, Tianjin, China. 2-Methylimidazole (C_4_H_6_N_2_, 98%) was obtained from Shanghai Macklin Biochemical Company Limited, Shanghai, China. Thiabendazole (C_10_H_7_N_3_S, AR, 98%) was procured from Shanghai Aladdin Biochemical Technology Limited Company, Shanghai, China. All other chemicals were supplied by Sinopharm Chemical Reagent Company Limited, Shanghai, China. All chemicals were used as received, without further purification.

### 3.2. Preparation of Thi@ZIF-8

**Figure 12:**
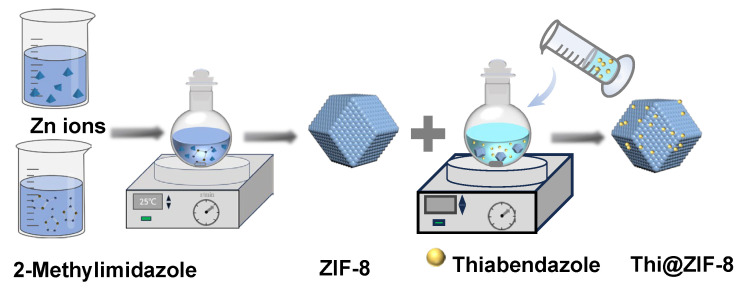
Preparation diagram of Thi@ZIF-8.

The preparation process of Thi@ZIF-8 is shown in Figure 12. Initially, a solution was prepared by dissolving 0.1800 g of Zn(CH_3_COO)_2_·2H_2_O in 15 mL of deionized water. Simultaneously, 2.4600 g of 2-methylimidazole was dissolved in another 15 mL of deionized water. The zinc nitrate solution was slowly poured into the 2-methylimidazole solution, ensuring thorough mixing with continuous stirring at room temperature. The resulting product was centrifuged at 10,000 rpm for 10 min. The subsequent washing steps were performed with absolute ethanol to ensure purity. The collected product was finally transferred to a drying oven and dried overnight at 53 °C, resulting in the formation of a white ZIF-8 powder.

Thi@ZIF-8 was prepared by adding 20 mL of Thi ethanol solution at different concentrations and 0.0400 g ZIF-8 while stirring. The methods used to centrifuge and dry the product were the same as those described above. Each treatment was performed in no less than six replicate series.

The loading efficiency of Thi on the Thi@ZIF-8 was determined by a UV–Vis spectrophotometer (Shanghai Yuanxi Instrument Company Limited, Shanghai, China) at a detection wavelength of 300 nm. Each treatment was performed within three replicate series. The loading rate of Thi was determined using Equation (1):

(1) $$\mathrm{Loading}\;\mathrm{rate}\;\%=\frac{\left(C_{0}-\left.C_{t}\right)V\right.}{\left(C_{0}-\left.C_{t}\right)V+M\right.}\times 100\%$$

where *C*_0_ and *C_t_* are the initial and residual concentrations (mg/mL) after time duration *t*, respectively. *M* is the weight (g) of ZIF-8 and *V* is the volume (mL) of the Thi solution.

### 3.3. Structural Characterization

The crystallinity was evaluated using X-ray diffraction (XRD; Rigaku Smartlab 3kw, Rigaku Corporation, Tokyo, Japan), operating with Cu Kα radiation and scanning a 2θ range of 5–50°. The chemical composition of the materials was validated using a Fourier-transform infrared spectrometer (FT-IR, Spectrum100, PERKINELMER, Waltham, MA, USA). Thermal gravimetric analysis (TGA, HITACHI STA200, HITACHI, Tokyo Metropolis, Japan) was used to record the weight loss data of the materials with temperature changes between 25 °C and 800 °C under an N_2_ atmosphere and a heating rate of 15 °C/min. The change in the Zeta potential of samples was obtained using a Zeta potential analyzer (2004, Malvern Instruments, Malvern, UK). The morphology and elemental distribution of the compounds were determined by a scanning electron microscope (SEM, ZEISS Sigma 300, Carl Zeiss AG, Oberkochen, Germany). The presence of elements and the interaction mechanisms were determined using X-ray photoelectron spectroscopy (XPS, Thermo Scientific K-Alpha, Waltham, MA, USA).

### 3.4. Thi Release Study

The dialysis bag method was used to investigate the release behavior of Thi@ZIF-8 under different pH conditions. Thi@ZIF-8 weighing 0.0200 g was packed into dialysis bags (8000–14,000 Mw), immersed in 100 mL of buffer (PBS/ethanol/Tween 80 = 90:9.5:0.5) solutions (in sink condition) with pH values of 5.0, 7.0, and 9.0 at 20 °C and 100 rpm r/min. The supernatant was collected in increments of 7.5 mL at regular intervals (the 7.5 mL of the original solution was replenished with fresh buffer solution immediately after each sampling) and analyzed using the UV–Vis method. At the same time, pH values were also detected to maintain the pH stability of the release buffer. All treatments were performed with three replicates. The cumulative release rate of Thi was determined using Equation (2):

(2) $$\mathrm{Cumulative}\;\mathrm{release}\;\%=\frac{V\sum_{i-0}^{n-1}C_{i}+V_{0}C_{n}}{M}\times 100\%$$

where *V* (7.5 mL) represents the volume of each sample extracted at different times, C*_n_* denotes the concentration of Thi (mg/mL) in the release solution at time n, V_0_ denotes the total volume of the release medium, and *M* denotes the total amount of Thi loaded.

### 3.5. Release Kinetics Mechanism

Four mathematical models were adopted to investigate the release mechanism, listed as follows:

$$\mathrm{Zero-order}\;\mathrm{model:}\;M_{t}/M_{\infty }=kt$$

$$\mathrm{First-order}\;\mathrm{model:}\;M_{t}/M_{\infty }=1-e^{-kt}$$

$$\mathrm{Higuchi}\;\mathrm{model:}\;M_{t}/M_{\infty }={kt}^{1/2}$$

$$\mathrm{Ritger}–\mathrm{Peppas}\;\mathrm{equation}\;\mathrm{model:}\;M_{t}/M_{\infty }={kt}^{n}$$

where *k*, *t*, *n*, and *M_t_/M*_∞_ represent the corresponding release kinetic constant, time, constant, and release ratio, respectively [65].

### 3.6. Column Leaching Performance

The test soil was collected from the top 0–15 cm of cultivated soil that had not been exposed to Thi for at least two years located in Jinzhong, China, and dried at room temperature. The debris was then removed from the collected soil samples. The samples were ground into small particles and filtered through 60 mesh sieves. The column leaching method was used [60,66].

The soil leaching experiments were carried out in a glass chromatographic column (diameter = 3.0 cm; length = 30 cm) packed with the sieved soil sample up to a height of 20 cm. Each column was pre-saturated with a certain amount of deionized water. When the adsorption of the soil in the column was fully balanced, 50 mg Thi@ZIF-8 was uniformly applied to the upper surface of the soil column, and the column was covered with a layer of filter paper. Subsequently, 150 mL of buffer (PBS/ethanol = 90:10 vol/vol) with different pH values (5.0, 7.0, and 9.0) were poured into the columns and the leachate was collected from the bottom of the columns.

After the leaching process was complete, the soil inside the column was divided into four equal segments (5 cm per segment). The total weight (Wt) of each section of the soil was measured. The soil in each section was mixed well by stirring, 2 g of the soil was transferred to a plastic centrifuge tube, and 10 mL ethanol and 2 μL concentrated hydrochloric acid were added. Thi was extracted from the above mixture by incubating it for 2 h and using an oscillator at 150 rpm/min at 25 °C. The mixture was centrifuged at 6000 rpm for 10 min and then filtered. The filtrate was saturated with sodium chloride, the Thi ethanol solution was precipitated, and the water layer was discarded. Ethanol was evaporated completely at 78.5 °C, and the solution was diluted to 5 mL with ethanol. Thi was detected by the UV–Vis method at a detection wavelength of 300 nm.

Simultaneously, the volume of the leachate from the soil column was measured. The leachate was then mixed thoroughly and the Thi in the leachate was measured using the same method described above.

For the control group, an experiment was conducted under the same conditions but using Thi instead of Thi@ZIF-8 and a buffer at pH 7.0. To account for handling errors, all experiments were performed in triplicate.

### 3.7. Bioactivity Study

1. Fungicidal activity in vitro

The mycelial growth rate method was used to determine the fungicidal activity against *B. cinerea*. Briefly, mycelial discs (8 mm in diameter) were placed on PDA media plates containing Thi or Thi@ZIF-8 (20 mg/L of Thi) and incubated at 25 ± 2 °C. Sterile water containing 0.1% Tween 80 was used as the control (CK), and each treatment was replicated three times. The colony diameters were measured using the crossover method three days after treatment. The inhibition rate was determined using Equation (3):

(3) $$\mathrm{Inhibition}\;\mathrm{rate}\;\%=\frac{D_{t}-D_{c}}{D_{c}-D_{0}}\times 100\%$$

where D*_t_* represents the colony diameter of each sample in the different treatments (mm), D*_c_* denotes the colony diameter of CK (mm), and D_0_ denotes the diameter of the mycelial discs (8 mm).

2. Fungicidal activity in vivo

Using sterilized inoculation needles to create an approximately 8 mm infection area on the surface of tomato fruits, 10 µL of Thi and Thi@ ZIF-8 solution (200 mg/L of Thi) was dripped onto the infection area, respectively. After the compounds had been completely absorbed, mycelial discs (8 mm in diameter) were placed on this area and incubated at 25 ± 2 °C. Sterile water containing 0.1% Tween 80 was used as the control. Each treatment was replicated three times. The colony diameters were measured using the crossover method seven days after treatment.

### 3.8. Tomato Plant Safety Evaluation

Tomato seeds were immersed in sterile water for 6 h and then taken out and wrapped in moist, sterile paper towels. Processed seeds were placed in Petri dishes in the dark. Tomato seeds were transplanted into pots after germination at 25 ± 2 °C. Each pot was filled with 30 g of dry soil, and water was added to maintain a humidity of approximately 70%. According to the Thi recommended concentrations of 1, 2, and 4 times, 130 mg/L, 260 mg/L, and 520 mg/L were used, respectively. After 24 h, 10 mL Thi of at various concentrations was sprayed in the pots, and water was used for the control group. Each treatment was repeated no less than three times. Plant height (mg), root length (mg), and fresh weight (mg) were measured after 7 and 14 d.

### 3.9. Statistical Analysis

Statistical analysis was performed with IBM SPSS v.19.0 software (IBM, Armonk, NY, USA). The data were processed using one-way ANOVA and a Duncan test (*p* < 0.05). All data are expressed as mean ± SD.

## 4. Conclusions

In this work, an eco-friendly and pH-responsive Thi@ZIF-8 was successfully developed using an easy preparation method for effectively controlling disease on tomatoes caused by *B. cinerea*. The preparation conditions of Thi@ZIF-8 were optimized, and the Thi@ZIF-8 was analyzed by different characterization techniques. Thi was loaded onto ZIF-8 and protected by the carrier, based on the results from SEM elemental mapping, XRD, FT-IR, and TGA. XPS data revealed that the binding energy of N, Zn, and S atoms in the sample changed after Thi loaded, which implied an interaction between Zn and Thi. The results of the in vitro release tests indicated that Thi@ZIF-8 displayed excellent pH-responsive release performance, and the release mechanism followed non-Fickian diffusion. Additionally, the fungicidal activity experiments demonstrated that Thi@ZIF-8 exhibited significantly enhanced control against *B. cinerea* compared with Thi in vitro. More importantly, the ZIF-8 carrier successfully restrained the released Thi from the top to the bottom of the soil column and had no side effects on tomato growth. In summary, the potential of Thi@ZIF-8 for improving the efficacy of pathogen control and attenuating environmental pollution risks may have promising application prospects in the sustainable development of modern agriculture and the production of cleaner products.

But, the current ZIF-8 carrier is relatively modest in improving the efficiency of Thi. Therefore, future research should focus on improving the performance of ZIF-8, which is critical for its application as an effective system for the smart protection of plants. First, while controlling the nanoparticle size, it is imperative to enhance the pesticide-loading capacity. Second, the precision and directional transport of pesticides are achieved by studying other release factors, such as temperature. Finally, enhancing the distribution and retention of droplets on foliar surfaces is crucial for its application. In addition, considering that ZIF-8 is a foreign substance in nature, to minimize potential risks, it is necessary to replace ZIF-8 with nature’s biological carriers or enhance preparation technological innovation, such as the self-assembly of precursor drug conjugates (SAPDC), nanochannel porous membranes, etc.

## Figures and Tables

**Table 1:** Kinetic parameters of different models for the release curve of Thi@ZIF-8.

Model	pH Value	*k*	*n*	R^2^
Zero-order	5.0	0.31		0.99212
	7.0	0.20		0.99752
	9.0	0.10		0.9654
First-order	5.0	0.0031		0.9972
	7.0	0.0021		0.99467
	9.0	0.0007		0.95011
Higuchi	5.0	5.52		0.97555
	7.0	3.50		0.96103
	9.0	1.73		0.90229
Ritger-Peppas	5.0	1.02	0.79	0.99874
	7.0	5.53	0.83	0.99728
	9.0	0.21	0.88	0.95458

**Table 2:** Inhibition rate of Thi and Thi@ZIF-8 against *B. cinerea*.

	Inhibition Rate on PDA/%	Inhibition Rate on Tomato/%
Thi	28.9 ± 1.83 b	22.76 ± 5.9 a
Thi@ZIF-8	59.07 ± 0.55 a	32.58 ± 5.8 a

Note: The data in the table are the average of 3 repetitions; Different letters in the same column indicate significant differences between treatments (*p* < 0.05).

## Data Availability

All experimental data acquired are reported in the manuscript.
